# The effect of terrain and female density on survival of neonatal white‐tailed deer and mule deer fawns

**DOI:** 10.1002/ece3.2178

**Published:** 2016-06-03

**Authors:** Maegwin Bonar, Micheline Manseau, Justin Geisheimer, Travis Bannatyne, Susan Lingle

**Affiliations:** ^1^ Department of BiologyUniversity of WinnipegWinnipegR3B 2E9ManitobaCanada; ^2^ Natural Resources InstituteUniversity of ManitobaWinnipegR3T 2N2ManitobaCanada; ^3^ Present address: Department of BiologyMemorial University of NewfoundlandSt. John'sNewfoundlandA1B3X9Canada

**Keywords:** Antipredator behavior, coyote predation, habitat use, NDVI, species interactions

## Abstract

Juvenile survival is a highly variable life‐history trait that is critical to population growth. Antipredator tactics, including an animal's use of its physical and social environment, are critical to juvenile survival. Here, we tested the hypothesis that habitat and social characteristics influence coyote (*Canis latrans*) predation on white‐tailed deer (*Odocoileus virginianus*) and mule deer (*O. hemionus*) fawns in similar ways during the neonatal period. This would contrast to winter when the habitat and social characteristics that provide the most safety for each species differ. We monitored seven cohorts of white‐tailed deer and mule deer fawns at a grassland study site in Alberta, Canada. We used logistic regression and a model selection procedure to determine how habitat characteristics, climatic conditions, and female density influenced fawn survival during the first 8 weeks of life. Fawn survival improved after springs with productive vegetation (high integrated Normalized Difference Vegetation Index values). Fawns that used steeper terrain were more likely to survive. Fawns of both species had improved survival in years with higher densities of mule deer females, but not with higher densities of white‐tailed deer females, as predicted if they benefit from protection by mule deer. Our results suggest that topographical variation is a critical resource for neonates of many ungulate species, even species like white‐tailed deer that use more gentle terrain when older. Further, our results raise the possibility that neonatal white‐tailed fawns may benefit from associating with mule deer females, which may contribute to the expansion of white‐tailed deer into areas occupied by mule deer.

## Introduction

The decline of many ungulate populations is widely attributed to poor juvenile survival, with predation identified as the primary proximate cause of mortality for juveniles (Hatter and Janz 1994; Pinard et al. 2012; Forrester and Wittmer 2013). Well‐known examples include mule deer (*Odocoileus hemionus*) and black‐tailed deer (*O. h. columbianus*) throughout western North America (Hatter and Janz 1994; Robinson et al. 2002; Cooley et al. 2008; Forrester and Wittmer 2013) and the more urgent case of caribou (*Rangifer tarandus*) throughout the north (Wittmer et al. 2005). The tactics that juveniles use to avoid predation, including their use of habitat features and protection provided by other individuals, are critical to juvenile survival. To better predict trends for ungulate populations, we must understand how the physical and social environment influences the risk of predation on juveniles.

Prey species that use different tactics to evade predators sometimes find safety in completely different habitats (Christensen and Persson 1993; Heithaus et al. 2009; Wirsing and Ripple 2011). Animals that are highly vulnerable to being captured when encountered may avoid areas rich in predators. Animals that effectively outdistance predators when encountered may reduce their overall risk of predation by living alongside their predators, rather than by moving into a different habitat with fewer predators where they face a decreased probability of escape (Lima 1992). For example, bottlenose dolphins (*Tursiops aduncus*) shift into habitats with tiger sharks (*Galeocerdo cuvier*) when these predators are abundant. This habitat gives dolphins ready access to deep water where they can outdistance and outmaneuver sharks (Heithaus et al. 2009; Wirsing et al. 2010). Pied cormorants (*Phalacrocorax varius*) and olive‐headed sea snakes (*Disteria major*) are less capable of avoiding capture when encountered and instead avoid areas where sharks are abundant.

The tactics that provide the most safety for neonates may differ from the tactics that provide safety to more mature animals. Females of many prey species appear to reduce the risk of predation facing their young by entering habitats with low predator abundance shortly before parturition (Edwards 1983; Bergerud et al. 1984). Some of these species live in hilly or mountainous terrain year‐round, pushing further into rugged or isolated terrain around the time of parturition (Bergerud et al. 1984; Festa‐Bianchet 1988; Bleich et al. 1997; Barten et al. 2001; Bangs et al. 2005; Long et al. 2009; Leclerc et al. 2012). Still other species, or members of a species, including elk (*Cervus elaphus*) (Mao et al. 2005) and pronghorn (*Antilocapra americana*) (Barnowe‐Meyer et al. 2010), occupy gentle terrain for most of the year, entering higher or steeper terrain at the time of parturition. The few studies that have measured the relationship between topography and neonatal survival report that neonatal ungulates are at less risk of wolf (*Canis lupus*) predation when using steeper terrain (caribou, Bergerud et al. 1984; pronghorn, Barnowe‐Meyer et al. 2010; caribou, Dussault et al. 2012), but may be at increased risk from other predators such as black bears (*Ursus americanus*) (Dussault et al. 2012).

The social environment also influences an animal's ability to evade predators. Prey are generally safer in large groups (Krause and Ruxton 2002), although the relationship between group size and safety can vary with the antipredator tactics used by a particular species (Lingle 2001). Antipredator costs associated with small groups or low population density may result in an Allee effect, reduced fitness at small population sizes (Gascoigne and Lipciu 2004; Courchamp et al. 2008). Similar to the physical environment, the social environment that provides the most safety can vary across species and across life stages. A juvenile may rely on conspecifics (Montgomerie and Weatherhead 1988; Cocroft 2002; Caro 2005) or even heterospecifics (Burger 1984; Lingle et al. 2005) to defend it from predators during the neonatal period, even if that same individual can successfully flee from predators after a few weeks or months of life.

Mule deer and white‐tailed deer (*O. virginianus*) are sister species that are similar in size (Mackie 1964), feeding habits (Anthony and Smith 1977), and reproductive behavior (Hirth 1977; Kucera 1978). They are sympatric throughout much of western North America. Within a geographic area, they breed and produce fawns at the same time of year (Whittaker and Lindzey 1999; Lingle et al. 2008). Differing antipredator tactics contribute to species differences in habitat and sociality during winter (Lingle 2002; Lingle and Pellis 2002). Mule deer stot when pursued by predators, and fawns are not fast enough by their first winter to outdistance coyotes across gentle terrain. Instead, they use steeper terrain to reduce their risk of being encountered or attacked (Lingle 2002). If encountered, fawns and adults bunch together with other deer, with females counterattacking coyotes to thwart attacks. In contrast, white‐tailed fawns gallop swiftly enough to outdistance coyotes in gentle terrain by their first winter (Lingle and Pellis 2002). White‐tailed fawns face much lower risk than mule deer fawns in gentle terrain during winter (Lingle 2002).

Mule deer females also use steep terrain when rearing young fawns, pushing further into rugged terrain before parturition (Long et al. 2009). Research into the relationship between habitat and survival of white‐tailed fawns has focused on characteristics of vegetation (Vreeland et al. 2004; Rohm et al. 2007; Grovenburg et al. 2011). The few descriptions of topography that are available suggest that white‐tailed females may occupy more rugged terrain during parturition than at other times of year (Wood et al. 1989; seasonal distribution maps in Lingle 2000; Lingle et al. 2005).

**Figure 1:**
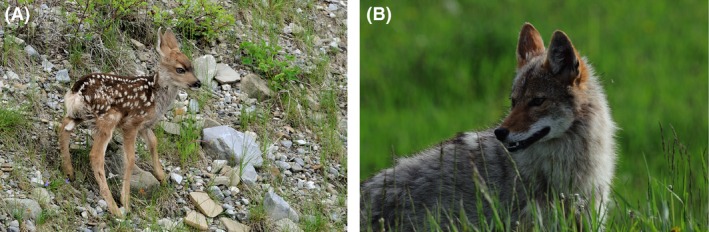
Mule deer fawn on steep terrain (A). Coyote (B) packs hunt mule deer and white‐tailed fawns, which are sympatric at the McIntyre Ranch, Alberta, Canada. Photographs © Peter Neuhaus.

Our aim in this study was to test the hypothesis that habitat and social characteristics influence survival of white‐tailed deer and mule deer fawns in similar ways during the neonatal period, in contrast to their divergent behavior in winter. The first prediction we tested was that mule deer and white‐tailed deer fawns living in steeper and more rugged terrain have improved survival, as predicted if both species use rugged terrain to reduce the risk of predation (Fig. 1A,B).

Our second prediction was that fawn survival would improve with increased densities of mule deer females, but not with increased densities of white‐tailed females. Mule deer females defend fawns that are not their own offspring, including white‐tailed fawns (Lingle et al. 2005). In fact, the simple presence of a mule deer female next to a fawn deters coyotes from approaching closer. We hypothesized that the protection provided by mule deer females would be impaired at low population densities, if their ability to deter coyotes from fawning areas is reduced when fewer females are available to congregate in fawning areas. Because of lower levels and less effective aggressive defense by white‐tailed females (Lingle et al. 2005), we did not expect to find a positive relationship between fawn survival and the density of white‐tailed females.

To examine effects of habitat and social traits on survival of neonates, it is necessary to control for environmental variation that affects survival across cohorts (Forchhammer et al. 2001). Winter and spring climatic conditions influence the productivity of vegetation (Pettorelli et al. 2005a). The quality of vegetation influences the quality of bed sites available to fawns, which may affect a fawn's ability to regulate its temperature (Van Moorter et al. 2009) or to avoid encounters with predators (Linnell et al. 1995; Shallow et al. 2015). The quality of vegetation also influences the physical condition of females and juveniles (Gaillard et al. 2000; Garel et al. 2011) and juvenile survival (Linnell et al. 1995; Gaillard et al. 2000; Hamel et al. 2010), both in areas with and without predators. Individuals that are in better physical condition should be better able to defend themselves or their offspring against predators (Lima and Dill 1990; Sinclair and Arcese 1995; Winnie and Creel 2006). To control for annual variation in environmental conditions, we included an index of vegetation productivity, the Normalized Difference Vegetation Index (NDVI), and weather conditions for the winter preceding birth of each cohort when testing the effect of terrain and female density on fawn survival.

## Materials and Methods

### Study site and subjects

**Figure 2:**
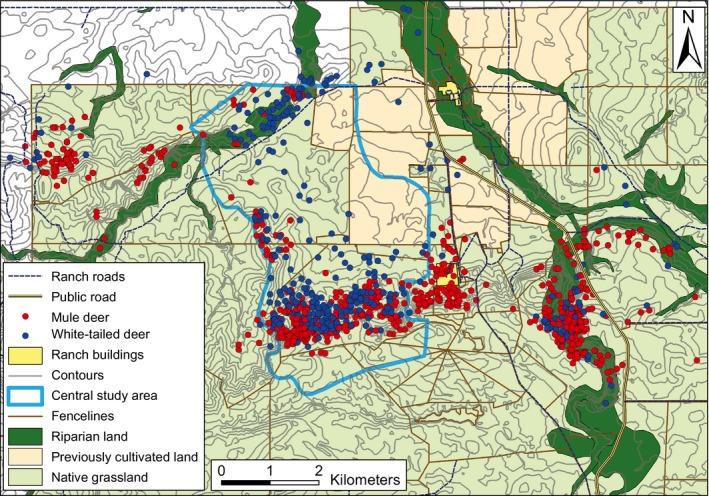
Map of the study area including locations of mule deer (red) and white‐tailed deer (blue) fawns.

We conducted research at the McIntyre Ranch, a 225‐km^2^ privately owned cattle ranch in southern Alberta, Canada (49°N, 112°W, elevation 1080–1380 m). The ranch has an open, rolling landscape dominated by fescue grassland (*Festuca scabrella*), with patches of short shrubs (0.5–2 m tall) including wild rose (*Rosa acicularis*), Saskatoon berry (*Amelanchier alnifolia*), and chokecherry (*Prunus virginiana*) in more mesic areas. The majority of white‐tailed deer and mule deer females raised their fawns along three slope systems formed by a prominent escarpment and two deep river valleys (Fig. 2). Coyotes were the only predator of fawns known to occupy the study site during this study, and they led to variable and often high levels of mortality on fawns during summer and winter (Lingle et al. 2008). Hunting of deer or coyotes by humans was not permitted inside the ranch at any time of year.

### Fawn capture and monitoring

We captured 131 white‐tailed fawns and 210 mule deer fawns in 7 years, with 99% of fawns captured in June. In 1994 and 1995, we captured fawns in a 20‐km^2^ portion of the ranch, which we refer to as the “central study area” (Fig. 2). We extended this area to a total of 50 km^2^ from 2000 to 2004 and to 60 km^2^ in 2005. We estimated a fawn's age in categories (<24 h, 1–2 days, 3–4 days, 5–7 days, 7–14 days) at capture using characteristics of mobility and coordination when active (observed before or during capture or after release), the extent of the freeze response, condition and color of hooves, condition of the umbilicus, and the fawn's size (Haugen and Speake 1959; Grovenburg et al. 2014). We expanded categories as needed (e.g., 2–5 days, 5–10 days) to increase the probability the fawn's age fell within the range. We assigned a birth date based on the median date within the age category and updated the age of the fawn at each subsequent sighting. We estimated 90% of both mule deer and white‐tailed deer to be less than 1 week in age at capture, with 28 of those fawns (21 mule deer, 7 white‐tailed) known to be newborn (<6 h). We estimated the remaining 21 mule deer and 13 white‐tailed fawns to be between 7 and 14 days in age. We attached colored plastic Allflex ear tags to all fawns for visual identification. In analyses reported here, we used data from sightings of each fawn until the fawn was estimated to be 8 weeks in age.

We used binoculars and spotting scopes to find marked fawns during intensive fieldwork within and outside the study site from June through August to spot marked individuals. Fieldwork included censuses of deer, searches for marked fawns, observation of coyote hunts, and other time‐intensive observations that increased our opportunities to see fawns. During each sighting, we recorded the fawn's location using Universal Transverse Mercator (UTM) coordinates to either a 100 × 100 m (1 ha) cell (1994 and 1995) or to a 50 × 50 m (0.25 ha) cell (2000–2005).

To improve our ability to monitor fawn survival, we attached 8 g VHF radio‐transmitters (www.holohil.com) with 12‐h mortality sensors to 72 of the white‐tailed fawns and 83 of the mule deer fawns we captured in 2000, 2001, 2004, and 2005 (see Lingle et al. 2008 for details). We tried two configurations (glue‐on transmitters and expandable collars) that remained attached for <1–6 weeks in 2000 and 2001. In 2004 and 2005, we attached transmitters with a loop antenna to a small ear tag (9.5 g for the transmitter and tag), which functioned successfully throughout the transmitter's 4‐month life span (see Lingle et al. 2008 for details). We attempted to get a signal from fawns with transmitters every day, searching more thoroughly for missing fawns typically every third day. We triangulated a fawn's location in seven cases when we had not observed the fawn for a few weeks. We include attack and kill locations only when we knew that coyotes initially attacked the fawn at the location, either based on our observing the predation event (9 attacks for fawns that survived and three that died) or after we used telemetry to find a kill site (*n *= 4). Clues surrounding most transmitters and carcass remains were insufficient to determine whether or not coyotes initially attacked the fawn at that location, and many carcass remains were cached underground or at a den.

We determined that coyote predation was the dominant cause of mortality for fawns during summer based on direct observation of predation events (Lingle et al. 2005), inspection of carcasses, and the sudden disappearance of fawns during the time periods when we observed coyotes actively hunting deer (Lingle 2000; Lingle et al. 2008). Of 36 fawns with ear‐tag radio‐transmitters that died during the first 12 weeks of life in 2004 and 2005, only 2–4 (5.5–11%) appeared to have died from a cause other than coyote predation (Lingle et al. 2008). We included fawns in the analysis when they appeared active and healthy during previous sightings and then disappeared suddenly with no indications of dispersal activity from the area. Although the cause of death for these fawns was unknown, we assume that most of these fawns died from coyote predation. Undoubtedly, some of these fawns died from other causes. We excluded three fawns that were known to have died from health issues from all analyses. Thirty‐five fawns disappeared during the first 3 weeks of life with us seeing the fawn only once or not at all. Because health issues were more likely to have been a larger cause of death at this early stage, we ran two analyses: one excluding these fawns (see Results) and one including them (see Table A1). Excluding these 35 fawns, we used 949 sightings for 197 mule deer fawns (164 mothers) and 393 sightings for 106 white‐tailed fawns (101 mothers) to examine how habitat, social, and climatic traits influenced their survival during the neonatal period.

### Habitat variables

We used Geographic Information Systems (GIS) software ArcMap 10 (ESRI, Redlands, CA, USA) and a digital elevation model to assess topographical features: use of slopes, steepness of terrain, ruggedness of terrain, and elevation. We used an existing outline of three slope systems within the study site to identify fawns that were on‐ or off‐slope. Steepness of terrain was the slope of a surface, measured in degrees from the horizontal. We aggregated the original 10 × 10 m cells that were generated for the raster layer into 50 × 50 m cells to measure the steepness or elevation for fawns captured between 2000 and 2005, or into 100 × 100 m cells for fawns captured in 1994 and 1995 (due to the different scale at which we recorded UTM in those years). We used the vector ruggedness measure (VRM) to depict the ruggedness of terrain. The VRM measures changes in aspect and slope, which generates an index of ruggedness that is independent of steepness of terrain (Hobson 1972; Sappington et al. 2007). We report average ruggedness across a 450 × 450 m neighborhood, after examining effects of ruggedness at different spatial scales.

We identified general vegetation type as one of three categories, outlined on existing maps: native grassland, riparian, and previously cultivated land that was reseeded in the 1980s as pasture containing native and exotic grasses and forbs (Fig. 2). We developed a raster layer of tall shrub cover by examining 25 × 25 m grid cells overlaid on air photographs (0.4 m resolution). We identified the location of patches of tall shrub, which usually consisted of Saskatoon berry (approximately 0.5–1.5 m in height) or chokecherry (approximately 1–2 m in height). Depending on the portion of the grid cell that contained shrub, it was assigned a “0” (no shrub), “1” (≤25% of cell had shrub), “2” (≤50% shrub), or “3” (>50% shrub). We used the median value for grid cells under a 100‐m‐diameter buffer surrounding the fawn's location. We also measured the distance of a fawn to riparian land.

We measured the distance of a fawn to anthropogenic areas using existing polygon layers representing ranch buildings that were currently in use (Fig. 2). Last, we measured the closest distance of a fawn to a coyote den using data on the position of coyote dens for years 1994, 1995, 2000, 2001, and 2005; we believe we identified all dens during intensive fieldwork with both coyotes and deer in the central study area in those 5 years.

### Female and fawn density

We used two methods to estimate the density of females and fawns within the 20‐km^2^ central study area. We used the average number of mule deer females counted in the central study area during winter (December–January of all 7 years) censuses as an indication of the density of mule deer females during the previous summer. We previously found that the number of mule deer females in this area was stable from summer to winter by censusing females during summer in 5 years (Lingle et al. 2008). We could not use this method for white‐tailed deer, because many white‐tailed deer entered the central study area before autumn. We used data on female density from the five summers (1994, 1995, 2000, 2001, and 2005) when we compared models including the density of white‐tailed females and mule deer females.

### Environmental conditions

We used NDVI values as a proxy for annual variation in the productivity of vegetation (Pettorelli et al. 2005b). These data were obtained for 1994–2005 with the Advanced Very High Resolution Radiometer sensor onboard National Oceanic and Atmospheric Administration satellites and processed by the Global Inventory Monitoring and Modeling Studies group (Tucker et al. 2005). We summed bimonthly values from mid‐April to the end of June to calculate the integrated spring NDVI (Pettorelli et al. 2005b; Hamel et al. 2009) during the period of green‐up.

Hourly and daily weather values were interpolated for our field site by Robert Bourchier, Agriculture and Agrifood Canada (robert.bourchier@canada.ca) using BioSim 10.3.1.2 (Régnière et al. 2014) and data from Environment Canada weather stations and Alberta Agriculture and Forestry AgroClimatic Information Service (http://agriculture.alberta.ca/acis/ [June 2014]). We used the daily values to calculate an average November through March value for temperature, precipitation, and wind speed for the winter preceding the birth of each cohort of fawns.

### Statistical analysis

The first step in the data analysis was to use a two‐way ANCOVA (JMP 7.0; SAS Institute 2007) to compare habitat traits used by fawns of the two species, also testing whether these habitat traits varied between fawns that lived or died. We included the fawn's age at each sighting as a covariate to determine whether habitat characteristics changed with age. We tested for interactions between species and survival and between age and species. We included the fawn's identity, nested within the mother's identity, as a random effect to control for the lack of independence between repeated sightings of the same individual or its twin (Hamel et al. 2009). We included birth year as a random effect to control for variation among cohorts. We transformed variables that did not meet assumptions of normality (ruggedness or VRM, cube root; distance to settled areas and association with tall shrub, square root).

We then used generalized estimating equations (GEE) and an information theoretic approach (Burnham et al. 2011) to compare models associated with the hypotheses that climate, terrain, or female density affected fawn survival. We used R for these analyses (R Development Core Team 2016). GEE models had a compound symmetric covariance structure and empirical standard errors, with a binomial distribution term and a logit link function for the response variable of fawn survival. We identified the model producing the lowest quasi‐likelihood under the independence criterion (QICu) (Hardin and Hilbe 2012) as the most predictive of the response variable being examined, choosing the simpler of two models if it fell within 2 QICu units of a more complex model (Arnold 2010; Mundry 2011). We examined odds ratios and confidence intervals for predictors in the highest ranked model to evaluate the direction and magnitude of relationship between predictors and response variables.

In the first comparison of models, we used data for fawns captured from across the 60‐km^2^ study area to compare three basic models: (1) terrain, (2) climate, and (3) terrain + climate. Although the different topographic variables (use of slopes, elevation, ruggedness, steepness) had a similar relationship to fawn survival, we included steepness in models because it reflected large‐scale variation in topography across the study area. We used the averaged value for steepness of terrain collected from different sightings for each fawn because the response variable (each fawn either lives or dies) did not enable us to include multiple observations for each fawn in this analysis. We tested two variations of the climate model. The first (climate: spring) included spring NDVI. The second (climate: spring + winter) included spring NDVI plus two weather variables, precipitation and wind speed from the preceding winter (November–March). These two variables were selected following a preliminary analysis of different weather variables, acknowledging that this aspect of the analysis constituted an exploratory data analysis. Last, we combined terrain and climate for the third model.

The variable “species” was included in all models because of large differences in the survival of white‐tailed and mule deer fawns during summer (Lingle et al. 2008). We included an interaction term involving the species of a fawn when it seemed likely that a predictor might have a different effect on fawns of the two species. We included the mother's identity as a random effect to control for the lack of independence between twins.

In a second comparison of models, we focused on fawns captured and living in the 20‐km^2^ central study area to examine the contribution of female density to models predicting fawn survival. We used female mule deer density over the 7 years to determine whether the addition of mule deer density improved the best model from the previous analysis. We also ran an analysis with a 5‐year data set for the central study area that enabled us to compare models that included either mule deer female density or white‐tailed female density.

We restricted the analysis to fawns <8 weeks in age because their habitats appeared relatively stable during this time, with no marked fawns dispersing to winter ranges until they were older (14–22 weeks in age). Nonetheless, the age of the fawn has the potential to confound results if surviving fawns changed habitats as they age. The inclusion of kill sites may also bias results to reflect the riskier portions of habitat for fawns that died, although this is unlikely to be an issue given the small number of kill sites in our data set. To ensure that the age of the fawn and the inclusion of kill sites did not bias results, we ran an additional analysis of models using data based on the locations where fawns were captured (see Table A1).

## Results

Across the seven summers, 63% of 129 white‐tailed fawns and 21.5% of 209 mule deer fawns died during the first 8 weeks of life. These rates decline to 55% of 106 white‐tailed fawns and 17% of 197 mule deer fawns (Lingle et al. 2008) when we exclude fawns that disappeared during the first 3 weeks of life for which we had insufficient evidence to assess a probable cause of death (see Materials and Methods).

### Species differences in habitat traits

**Figure 3:**
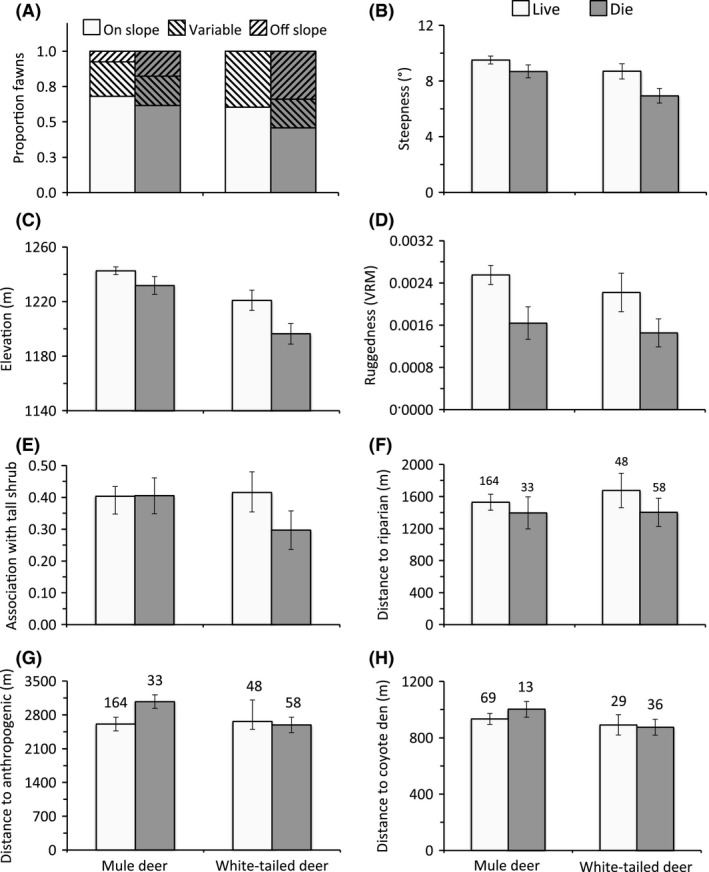
Relationship between species of fawn, survival, and habitat traits for white‐tailed deer and mule deer fawns: (A) use of slopes, (B) steepness of terrain, (C) elevation, (D) ruggedness of terrain (VRM), (E) association with tall shrub, (F) distance to riparian areas, (G) distance to anthropogenic features, and (H) distance to closest coyote den. Panel (A) shows the average proportion of sightings in each type of terrain, using one value for each fawn. Panels (B–H) show the mean ± SE for each habitat trait, using an average value for each fawn. For tall shrub, 0 = no tall shrub in buffer of 100 m diameter around fawn; 1 = < 25% of the buffer was covered by shrub. The sample for panels (A) through (G) includes 197 mule deer (164 lived, 33 died) and 106 white‐tailed deer (48 lived, 58 died) (*n* shown on panels F, G). The sample for panel (H) includes 82 mule deer (69 lived and 13 died) and 65 white‐tailed (29 lived and 36 died) fawns that lived in the central study area.

Mule deer fawns were more likely to occupy slopes, higher elevations, and steeper terrain than were white‐tailed deer fawns (Fig. 3A–C; Table 1). There was no difference between the ruggedness of their terrain (Fig. 3D; Table 1). These four characteristics of terrain were related to survival in similar ways for the two species. Fawns that survived were more likely to have occupied slopes, higher elevations, steeper and more rugged terrain.

**Table 1:** Parameter estimates for two‐way ANCOVA to examine relationship between species, survival outcome, and habitat traits for mule deer and white‐tailed deer fawns while controlling for the age of the fawn. The birth year and the fawn's identity, nested within the mother's identity, were included as random factors. Results for most habitat variables are based on 1342 observations for 197 mule deer and 106 white‐tailed fawns monitored over seven summers. Results for distance to nearest coyote den were restricted to 82 mule deer and 65 white‐tailed fawns (813 sightings) living in the central study area in five summers

Habitat characteristic	Species^a^		Survival		Age	
	*F*	*P*	*F*	*P*	*F*	*P*
Elevation	22.589	<0.001	8.700	0.003	22.002	<0.001
Steepness	12.212	<0.001	10.369	0.001	7.589	0.006
VRM	1.163	0.282	3.728	0.054	1.474	0.225
Use of slopes	6.941^a^	0.008	6.459^a^	0.011	0.178^a^	0.674
Distance to riparian	0.024	0.877	0.045	0.832	32.191	<0.001
Tall shrub	1.948	0.164	0.079	0.779	1.807	0.179
Distance to anthropogenic features	0.014	0.905	0.337	0.562	0.150	0.700^b^
Distance to coyote den	2.369	0.126	<0.001	0.985	4.429	0.036

VRM, vector ruggedness measure.

a *F*‐scores and *P*‐values reported for all habitat traits except for use of slopes, for which we report Wald chi‐square value and *P*‐value. df = 1 for all variables.

b We tested the interactions Species*Survival and Species*Age for all traits. Age*Species was significantly associated with the distance to anthropogenic features (*F* = 20.151, *P *< 0.001), with mule deer fawns moving closer to, and white‐tailed fawns further from, anthropogenic areas as they aged. No other interactions were significant.

Fawns were nearly always associated with native vegetation (100% of mule deer and >99% of white‐tailed sightings), even though 17% percent of the study area had been modified because of previous cultivation and subsequent reseeding of the pasture (Fig. 2). We therefore could not assess the relationship between the general type of vegetation and survival. Other habitat traits including the association with tall shrub, the distance to riparian areas or anthropogenic areas, and the distance to the nearest coyote den did not differ significantly between the species and were not related to fawn survival (Fig. 3E–H; Table 1).

Age did not affect the fawns' use of slopes over the 8‐week time period, but it was related to some habitat traits (Table 1). Older fawns used terrain that was lower in elevation and less steep, closer to coyote dens, and closer to riparian areas. We detected one interaction between age and species, with mule deer fawns moving closer to, and white‐tailed fawns moving further from, anthropogenic areas as they aged.

### Habitat, social, and climatic factors affecting fawn survival

A model including steepness of terrain and spring/winter climate was the most parsimonious model explaining survival of fawns during the first 8 weeks of life, with no interaction between species and steepness of terrain (Table 2). This was true when we ran models with averaged habitat data from different sightings (Table 2), with data from capture locations alone (Table A1), or with data including fawns that disappeared during the first 3 weeks, for which we had insufficient information to assign a probable cause of death (Table A2). Fawns living in steeper terrain were more likely to survive (Table 3, odds ratio, 5th – 95th CI = 1.156, 1.043–1.280, Wald *χ* ^2^ = 7.696, *P* = 0.006; Table A3). Fawns had improved survival in years with a higher integrated spring NDVI (Table 3; Table A3).

**Table 2:** Model selection results for a priori climate and terrain models of survival for 197 mule deer and 106 white‐tailed deer fawns at the McIntyre Ranch, Alberta, Canada, over 7 years (1994, 1995, 2000, 2001, and 2003–2005)

Model	Predictors	*k*	QICu	ΔQICu	wi
Null model	Intercept	1	374.82	96.57	0.00
Species	Species	2	331.61	53.36	0.00
1A. Terrain	Species + Steepness	3	326.39	48.14	0.00
1B. Terrain	Species + Steepness + Species*Steepness	4	328.39	50.14	0.00
2A. Climate (spring)	Species + Spring NDVI	3	303.72	25.47	0.00
2B. Climate (spring & winter)	Species + Spring NDVI + Winter ppt + Winter wind speed	5	286.83	8.58	0.01
3A. Terrain + Climate (spring)	Species + Steepness + Spring NDVI	4	294.18	15.93	0.00
3B. Terrain + Climate (spring & winter)	Species + Steepness + Spring NDVI + Winter ppt + Winter wind speed	6	278.25*	0.00	0.99

We used the averaged value for habitat characteristics based on different sightings for each fawn. For each model, we report the quasi‐likelihood under the independence criterion (QICu), the deviation from the lowest QICu score (ΔQICu), and model weight (wi). QICu value marked with an asterisk indicates the model with the strongest support. Species was included in all models because of large differences in survival during this stage of their lives.

NDVI, integrated spring Normalized Difference Vegetation Index; ppt, precipitation.

**Table 3:** Parameter estimates for best‐supported model (Table 2, Model 3B) for fawn survival that considers terrain and climate variables for 197 mule deer (MD) fawns and 106 white‐tailed deer (WT) fawns at the McIntyre Ranch, Alberta, Canada, over 7 years (1994, 1995, 2000, 2001, and 2003–2005)

Variable	df	Odds ratio	CI (5th, 95th) of odds ratio		Wald *χ* ^2^	*P*
Species (MD/WT)	1	5.817	3.050	11.094	28.569	<0.001
Steepness (°)	1	1.156	1.043	1.280	7.696	0.006
NDVI	1	1.006	1.003	1.009	14.207	<0.001
Winter ppt (mm)	1	1.072	1.000	1.149	3.886	0.049
Winter wind speed (km/h)	1	0.602	0.391	0.926	5.346	0.021

NDVI, integrated spring Normalized Difference Vegetation Index; ppt, precipitation.

The addition of mule deer female density improved the model explaining fawn survival in the central study area using the averaged habitat data (Table 4) or data from capture locations (Table A4). Mule deer density was highly correlated with winter precipitation (*r *= 0.839). The addition of mule deer density to a model with species, spring NDVI, and steepness improved the model more than the addition of winter climatic variables (Tables 4, A4). Steepness, spring NDVI, and mule deer density were influential predictors (Tables 5, A5).

**Table 4:** Model selection results for a priori climate, terrain, and mule deer (MD) female density models of survival for 93 mule deer and 86 white‐tailed deer fawns living in the central study area at the McIntyre Ranch, Alberta, Canada, over 7 years (1994, 1995, 2000, 2001, and 2003–2005)

Model	Predictors	*k*	QICu	ΔQICu	wi
Null model	Intercept	1	234.26	80.04	0.00
Species	Species	2	196.19	41.97	0.00
1A. Terrain + Climate (spring)	Species + Steepness + Spring NDVI	4	169.85	15.63	0.00
1B. Terrain + Climate (spring & winter)	Species + Steepness + Spring NDVI + Winter ppt + Winter wind speed	6	161.70	7.48	0.01
2A. Mule deer female density	Species + MD density	3	176.09	21.87	0.00
2B. Mule deer female density	Species + MD density + MD density*Species	4	175.17	20.95	0.00
3A. Terrain + Climate (spring) + Mule deer female density	Species + Steepness + Spring NDVI + MD density	5	155.95*	1.73	0.24
3B. Terrain + Climate (spring) + Mule deer female density	Species + Steepness + Spring NDVI + MD density + (Species*MD density)	6	154.22	0.00	0.57
3C. Terrain + Climate (spring & winter) + Mule deer female density	Species + Steepness + Spring NDVI + Winter ppt + Winter wind speed + MD density	7	158.99	4.77	0.05
3D. Terrain + Climate (spring & winter) + Mule deer female density	Species + Steepness + Spring NDVI + Winter ppt + Winter wind speed + MD density + (Species*MD density)	8	157.23	3.01	0.13

We used the averaged value for habitat characteristics based on different sightings for each fawn. For each model, we report the quasi‐likelihood under the independence criterion (QICu), the deviation from the lowest QICu score (ΔQICu), and model weight (wi). QICu values marked with an asterisk indicate competing models with the strongest support. Species was included in all models because of large differences in survival during this stage of their lives.

NDVI, integrated spring Normalized Difference Vegetation Index; ppt, precipitation.

**Table 5:** Parameter estimates for best‐supported models for fawn survival that considers density of mule deer females in addition to terrain and climate for 93 mule deer (MD) fawns, and 86 white‐tailed deer (WT) fawns living in the central study area at the McIntyre Ranch, Alberta, Canada, over 7 years (1994, 1995, 2000, 2001, and 2003–2005)

Model	Variable	df	Odds ratio	CI (5th, 95th) of odds ratio		Wald *χ* ^2^	*P*
3A. Terrain + Climate (spring) + Mule deer density	Species (MD/WT)	1	6.303	2.647	15.009	12.738	<0.001
	Steepness (°)	1	1.162	1.039	1.299	4.895	0.027
	NDVI	1	1.004	1.002	1.006	12.531	<0.001
	Mule deer density (no./km^2^)	1	1.044	1.017	1.071	12.838	<0.001
3B. Terrain + Climate (spring) + Mule deer density (including interaction with species of fawn)	Species (MD/WT)	1	33.185	3.726	295.584	9.750	0.002
	Steepness (°)	1	1.151	1.023	1.295	4.040	0.044
	NDVI	1	1.004	1.002	1.007	11.324	0.001
	Mule deer density (no./km^2^)	1	1.014	0.980	1.049	0.899	0.343
	Species * MD density (no./km^2^)	1	1.048	0.991	1.108	3.171	0.075

NDVI, integrated spring Normalized Difference Vegetation Index; ppt, precipitation.

**Figure 4:**
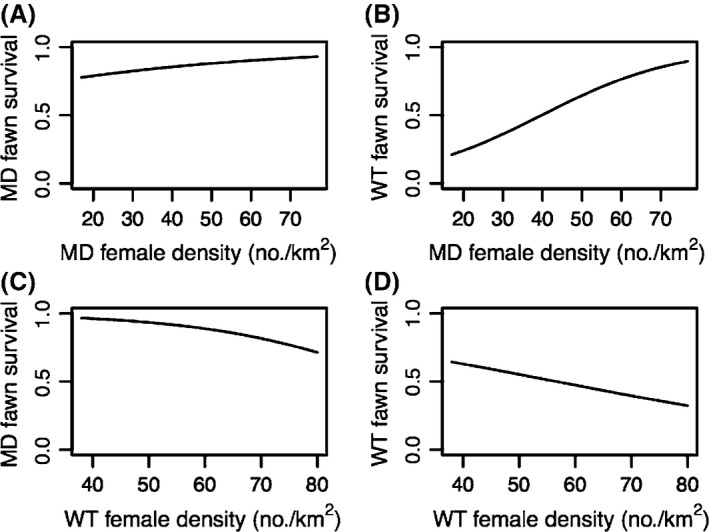
The probability of survival (1.0 = survival) for fawns over the first 8 weeks of life relative to annual variation in female density. Survival of (A) 93 mule deer (MD) and (B) 86 white‐tailed deer (WT) fawns relative to the density of mule deer females in 7 years. Survival of (C) 82 mule deer and (D) 65 white‐tailed fawns relative to the density of white‐tailed females in 5 years.

The density of mule deer females had a positive relationship with fawn survival (Table 5, odds ratio, 5th to 95th CI = 1.044, 1.017–1.071, Wald *χ* ^2^ = 12.838, *P* < 0.001; Table A5). The relationship between mule deer female density and survival of white‐tailed fawns appeared stronger than the relationship between mule deer female density and survival of mule deer fawns (Fig. 4A,B). Addition of the interaction term reduced the QICu by <2 (models 3A vs. 3B in Tables 4, A4), which suggests this model is an unsupported embellishment of the simpler model (Arnold 2010; Mundry 2011). We had a small sample of marked mule deer fawns (*n *< 5) in the central study area in the 2 years when mule deer females were lowest in density, which may have made it difficult to detect the contribution of the interaction.

In contrast to the density of mule deer females, the addition of white‐tailed female density did not improve models having terrain and climatic variables (Table A6). The density of white‐tailed females did not have a positive relationship with survival of mule deer or white‐tailed deer fawns (Fig. 4C,D) and was not an influential predictor when added to models instead of mule deer density (e.g., Table A6, model 4A, odds ratio, 5th – 95th CI for white‐tailed density: 0.984, 0.942–1.027, Wald *χ* ^2^ = 0.564, *P* = 0.453).

## Discussion

Although mule deer fawns had better survival compared with white‐tailed deer fawns, habitat and social variables influenced survival of fawns from both species in similar ways. Fawns of both species had improved survival when they lived in steeper terrain. Fawn survival also appeared to improve with increased mule deer female densities. When combined with the literature (Lingle 2002), these results indicate that mule deer fawns are safest from coyote predation in steeper terrain year‐round. Our results for white‐tailed fawns, a species that inhabits gentle terrain during winter, suggest that the habitat that is safest for this species varies with the life stage of the animal.

Mule deer occupy relatively rugged terrain throughout the year (Swenson et al. 1983; Wiggers and Beasom 1986; Wood et al. 1989; Lingle 2002) and are reported to use even steeper terrain following parturition (Long et al. 2009). Few studies – and none to our knowledge for mule deer – have measured the effect of terrain on neonatal survival, even though many species shift into steeper and more rugged terrain during parturition (see Introduction).

In contrast to mule deer, white‐tailed deer at this location inhabit gentle rolling terrain rather than slopes during winter (Swenson et al. 1983; Wiggers and Beasom 1986; Wood et al. 1989; Lingle 2002). Although mule deer neonates typically used higher elevations and slightly steeper terrain than white‐tailed neonates, species differences in terrain during summer were small compared with winter. Our findings for white‐tailed deer, and previous work on habitat selection by elk (Mao et al. 2005) and neonatal survival of pronghorn (Barnowe‐Meyer et al. 2010), highlight the need to consider topographical variation as a potentially critical resource for neonates of many ungulate species, even species like white‐tailed deer that may not use steep terrain when animals are older.

Fawns of both species were more likely to use lower elevations and less steep terrain as they aged, although they did not reduce their use of slopes during this 8‐week period. Sightings of fawns that survived inevitably included more sightings of older fawns. Nonetheless, we found that fawns that survived were more likely to have used higher and steeper terrain than fawns that died. We obtained this result when using averaged values for habitat data from repeated sightings of fawns or habitat values from locations where we captured fawns, which were usually (90% of 338 fawns) less than 1 week in age.

The general view is that females move from risky to safer habitats around parturition (Festa‐Bianchet 1988; Bleich et al. 1997). Another equally plausible interpretation is that the habitat that provides the most safety differs with the life stage of the animal and the tactics it uses at different life stages. If an immature animal has a small chance of avoiding capture when encountered, it may be safest by occupying a habitat where encounters are rare (Lima 1992). If an older animal is readily able to outdistance a predator over gentle terrain, it may lower its overall risk of predation despite accepting a high risk of encounter (e.g., Lima 1992; Wirsing et al. 2010). Because of their ability to outrun many predators, pronghorn, elk, and white‐tailed deer may be at less absolute risk in gentle terrain than in rugged terrain after they reach a certain age, rather than simply capable of tolerating additional risk.

We did not detect a relationship between survival and an animal's association with tall shrub for either species. However, tall shrub is uncommon at this location. Probably more important to fawns during the hiding stage is the density of low vegetative cover (forbs and grass) to conceal bedded fawns from predators (Barrett 1981; Van Moorter et al. 2009; Grovenburg et al. 2010; Shallow et al. 2015), a variable that we did not evaluate during this project.

Survival of neonates did not decline with increasing densities of mule deer females, as expected from traditional density dependent effects of competition for food or space (Gaillard et al. 1998). On the contrary, fawn survival appeared to improve in summers with higher densities of mule deer females, but not with higher densities of white‐tailed deer females. Longer term data are needed to better distinguish the effect of female density from climatic conditions that vary annually. Nonetheless, these results are consistent with the previous finding that mule deer females – and not white‐tailed females – protect neonatal fawns other than their own offspring, including white‐tailed deer fawns (Lingle et al. 2005).

We hypothesize that larger numbers of mule deer females congregate in fawning areas when their population is at higher density, improving their ability to deter coyotes and to defend fawns. White‐tailed females rear their fawns in many of the same areas as do mule deer (Fig. 2), and they should have increased opportunity to remain near mule deer females when mule deer are higher in density. To test these hypotheses, we need to examine how the local distribution of mule deer females and the outcome of their encounters with coyotes change with the density of the mule deer population.

These findings may shed light on a current dynamic facing white‐tailed deer and mule deer in the west. There is widespread concern with the expansion of white‐tailed populations in portions of western North America where mule deer populations are declining (Forrester and Wittmer 2013). Our results suggest that neonatal white‐tailed deer may benefit from this association with mule deer, which may contribute to the expansion of white‐tailed deer into areas occupied by mule deer.

Juveniles must survive their first summer if they are to survive their first year of life. A less obvious point is that high levels of survival during summer do not automatically translate to high levels of annual survival. First, climatic conditions that improve survival early in life may not necessarily improve survival over the full year. In our system, we cannot assume that high NDVI and high rates of fawn survival during summer translate to a high rate of annual survival. For example, the two summers (1994 and 2003) having the highest NDVI (results shown here) and high rates of fawn survival were followed by winters in which fawns suffered high rates of predation, reaching 99% in the case of mule deer fawns (Lingle et al. 2008). These observations are consistent with a report of increased winter mortality of mule deer fawns following springs with higher NDVI values (Hurley et al. 2014).

Second, conditions occurring during a particular season may have a larger influence on annual survival for one species than for another (Lingle et al. 2008). Although mule deer fawns are less vulnerable than white‐tailed fawns to coyotes during the initial months of life (Whittaker and Lindzey 1999; Lingle 2000), they are more vulnerable to coyote predation at our field site during winter (Lingle and Pellis 2002). As a result, variation in predation rates during winter is the primary factor shaping annual predation rates for mule deer fawns at this location.

Conversely, the level of predation occurring during summer is the primary factor shaping annual predation rates for white‐tailed fawns at this location (Lingle et al. 2008). Conditions that enable white‐tailed fawns to survive this seasonal bottleneck should therefore have a more direct relationship with their annual survival rates than is the case for mule deer. The availability of steeper terrain and an association with mule deer females have the potential to improve both summer and annual rates of survival for white‐tailed deer fawns.

## Conflict of Interest

None declared.

## Data Accessibility

Data available from the Dryad Digital Repository: http://dx.doi.org/10.5061/dryad.bg04r.

## Appendix

**Table A1:** Model selection results for a priori climate and terrain models of survival for 197 mule deer and 106 white‐tailed deer fawns at the McIntyre Ranch, Alberta Canada based on their capture locations over seven years (1994, 1995, 2000, 2001, 2003‐2005). For each model, we report the quasi‐likelihood under the independence criterion (QICu), the deviation from the lowest QICu score (ΔQICu), and model weight (wi). QICu value marked with an asterisk indicates the model with the strongest support. Species was included in all models because of large differences in survival during this stage of their lives. NDVI = integrated spring Normalized Difference Vegetation Index; ppt = precipitation

Model	Predictors	k	QICu	ΔQICu	wi
Null model	Intercept	1	374.81	98.92	0.00
Species	Species	2	331.61	55.72	0.00
1A. Terrain	Species + Steepness	3	322.08	46.19	0.00
1B. Terrain	Species + Steepness + Species*Steepness	4	323.67	47.78	0.00
2A. Climate (spring)	Species + Spring NDVI	3	303.72	27.83	0.00
2B. Climate (spring & winter)	Species + Spring NDVI + Winter ppt + Winter wind speed	5	286.82	10.93	0.00
3A. Terrain + Climate (spring)	Species + Steepness + Spring NDVI	4	289.92	14.03	0.00
3B. Terrain + Climate (spring & winter)	Species + Steepness + Spring NDVI + Winter ppt + Winter wind speed	6	275.89*	0.00	0.99

**Table A2:** Model selection results for a priori climate and terrain models of survival for 209 mule deer and 129 white‐tailed deer fawns at the McIntyre Ranch, Alberta Canada over seven years (1994, 1995, 2000, 2001, 2003‐2005). We used the averaged value for habitat characteristics based on different sightings for each fawn, and include data for 35 fawns that disappeared early for which we were unable to assess a probable cause of death. For each model, we report the quasi‐likelihood under the independence criterion (QICu), the deviation from the lowest QICu score (ΔQICu), and model weight (wi). QICu value marked with an asterisk indicates the model with the strongest support. Species was included in all models because of large differences in survival during this stage of their lives. NDVI = integrated spring Normalized Difference Vegetation Index; ppt = precipitation

Model	Predictors	k	QICu	ΔQICu	wi
Null model	Intercept	1	450.50	105.26	0.00
Species	Species	2	395.09	49.85	0.00
1A. Terrain	Species + Steepness	3	393.61	48.37	0.00
1B. Terrain	Species + Steepness + Species*Steepness	4	395.44	50.20	0.00
2A. Climate (spring)	Species + Spring NDVI	3	364.30	19.06	0.00
2B. Climate (spring & winter)	Species + Spring NDVI + Winter ppt + Winter wind speed	5	348.97	3.73	0.13
3A. Terrain + Climate (spring)	Species + Steepness + Spring NDVI	4	360.54	15.30	0.00
3B. Terrain + Climate (spring & winter)	Species + Steepness + Spring NDVI + Winter ppt + Winter wind speed	6	345.24*	0.00	0.87

**Table A3:** Parameter estimates for best‐supported model for fawn survival (from Table A1) that considers terrain and climate variables for 197 mule deer (MD) fawns and 106 white‐tailed deer (WT) fawns at the McIntyre Ranch, Alberta Canada based on their capture locations over seven years (1994, 1995, 2000, 2001, 2003‐2005). NDVI = integrated spring Normalized Difference Vegetation Index

Variable	df	Odds ratio	CI (5^th^, 95^th^) of odds ratio		Wald *χ* ^2^	*P*
Species (MD/WT)	1	6.570	3.522	12.256	35.026	<0.001
Steepness (°)	1	1.134	1.052	1.223	10.670	0.001
NDVI	1	1.005	1.003	1.008	15.625	<0.001
Winter precipitation (mm)	1	1.071	1.000	1.147	3.847	0.050
Winter wind speed (km/h)	1	0.619	0.407	0.942	5.021	0.025

**Table A4:** Model selection results for a priori climate, terrain and mule deer (MD) female density models of survival for 93 mule deer and 86 white‐tailed deer fawns living in the central study area at the McIntyre Ranch, Alberta Canada based on their capture locations over seven years (1994, 1995, 2000, 2001, 2003‐2005). For each model, we report the quasi‐likelihood under the independence criterion (QICu), the deviation from the lowest QICu score (ΔQICu), and model weight (wi). QICu value marked with an asterisk indicate the model with the strongest support. Species was included in all models because of large differences in survival during this stage of their lives. NDVI = integrated spring Normalized Difference Vegetation Index; ppt = precipitation

Model	Predictors	k	QICu	ΔQICu	wi
Null model	Intercept	1	234.28	80.90	0.00
Species	Species	2	196.19	42.80	0.00
1A. Terrain + Climate (spring)	Species + Steepness + Spring NDVI	4	166.57	13.18	0.00
1B. Terrain + Climate (spring & winter)	Species + Steepness + Spring NDVI + Winter ppt + Winter wind speed	6	160.30	6.92	0.02
2A. Mule deer female density	Species + MD density	3	176.09	22.71	0.00
2B. Mule deer female density	Species + MD density + MD density*Species	4	175.16	21.78	0.00
3A. Terrain + Climate (spring) + Mule deer female density	Species + Steepness + Spring NDVI + MD density	5	154.66*	1.28	0.29
3B. Terrain + Climate (spring) + Mule deer female density	Species + Steepness + Spring NDVI + MD density + (Species*MD density)	6	153.38	0.00	0.54
3C. Terrain + Climate (spring & winter) + Mule deer female density	Species + Steepness + Spring NDVI + Winter ppt + Winter wind speed + MD density	7	158.06	4.68	0.05
3D. Terrain + Climate (spring & winter) + Mule deer female density	Species + Steepness + Spring NDVI + Winter ppt + Winter wind speed + MD density + (Species*MD density)	8	156.72	3.33	0.10

**Table A5:** Parameter estimates for best‐supported model for fawn survival (from Table A4) that considers terrain, climate and the density of mule deer females for 93 mule deer (MD) fawns and 86 white‐tailed deer (WT) fawns in the central study area at the McIntyre Ranch, Alberta Canada based on their capture locations over seven years (1994, 1995, 2000, 2001, 2003‐2005). NDVI = integrated spring Normalized Difference Vegetation Index

Model	Variable	df	Odds ratio	CI (5^th^, 95^th^) of odds ratio		Wald *χ* ^2^	*P*
3A. Terrain + Climate (spring) + Mule deer density	Species (MD/WT)	1	5.910	2.747	12.716	17.300	<0.001
	Steepness (°)	1	1.102	0.998	1.215	6.965	0.008
	NDVI	1	1.003	1.002	1.005	13.511	<0.001
	MD density (no./km^2^)	1	1.034	1.011	1.057	10.830	0.001
3B. Terrain + Climate (spring) + Mule deer density (including interaction with species of fawn)	Species (MD/WT)	1	12.973	2.045	82.312	9.852	0.002
	Steepness (°)	1	1.094	0.989	1.210	5.464	0.019
	NDVI	1	1.003	1.002	1.005	11.730	0.001
	MD density (no./km^2^)	1	1.020	0.986	1.055	0.665	0.415
	Species * MD density (no./km^2^)	1	1.022	0.975	1.070	2.714	0.099

**Table A6:** Model selection results for a priori climate, terrain and mule deer (MD) or white‐tailed deer (WT) female density models of survival for 82 mule deer and 65 white‐tailed deer fawns living in the central study area at the McIntyre Ranch, Alberta Canada over five summers (1994, 1995, 2000, 2001, 2005). We used the averaged value for habitat characteristics based on different sightings for each fawn. For each model, we report the quasi‐likelihood under the independence criterion (QICu), the deviation from the lowest QICu score (ΔQICu), and model weight (wi). QICu value marked with an asterisk indicates the model with the strongest support. Species was included in all models because of large differences in survival during this stage of their lives. NDVI = integrated spring Normalized Difference Vegetation Index; ppt = precipitation

Model	Predictors	k	QICu	ΔQICu	wi
Null model	Intercept	1	189.14	53.44	0.00
Species	Species	2	165.08	29.38	0.00
1A. Terrain + Climate (spring)	Species + Steepness + Spring NDVI	4	143.33	7.63	0.01
1B. Terrain + Climate (spring & winter)	Species + Steepness + Spring NDVI + Winter ppt + Winter wind speed	6	136.95	1.25	0.15
2A. Mule deer female density	Species + MD density	3	142.64	6.94	0.01
2B. Mule deer female density	Species + MD density + MD density*Species	4	142.97	7.27	0.01
3A. Terrain + Climate (spring) + Mule deer female density	Species + Steepness + Spring NDVI + MD density	5	135.70*	0.00	0.28
3B. Terrain + Climate (spring) + Mule deer female density	Species + Steepness + Spring NDVI + MD density + (Species*MD density)	6	136.22	0.52	0.22
3C. Terrain + Climate (spring & winter) + Mule deer female density	Species + Steepness + Spring NDVI + Winter ppt + Winter wind speed + MD density	7	137.96	2.26	0.09
3D. Terrain + Climate (spring & winter) + Mule deer female density	Species + Steepness + Spring NDVI + Winter ppt + Winter wind speed + MD density + (Species*MD density)	8	138.05	2.35	0.09
4A. Terrain + Climate (spring) + White‐tailed deer female density	Species + Steepness + Spring NDVI + WT density	5	144.74	9.04	0.00
4B. Terrain + Climate (spring) + White‐tailed deer female density	Species + Steepness + Spring NDVI + MD density + (Species*WT density)	6	146.46	10.76	0.00
4C. Terrain + Climate (spring & winter) + White‐tailed deer female density	Species + Steepness + Spring NDVI + Winter ppt + Winter wind speed + WT density	7	137.96	2.26	0.09
4D. Terrain + Climate (spring & winter) + White‐tailed deer female density	Species + Steepness + Spring NDVI + Winter ppt + Winter wind speed + (Species*WT density)	8	139.17	3.47	0.05
